# Update on Tenosynovial Giant Cell Tumor, an Inflammatory Arthritis With Neoplastic Features

**DOI:** 10.3389/fimmu.2022.820046

**Published:** 2022-02-21

**Authors:** Marie Robert, Helena Farese, Pierre Miossec

**Affiliations:** Department of Clinical Immunology and Rheumatology, and Immunogenomics and Inflammation Research Unit, University of Lyon, Hôpital Edouard Herriot, Lyon, France

**Keywords:** tenosynovial giant cell tumor, pigmented villonodular synovitis, rheumatoid arthritis, sarcoma, targeted therapies

## Abstract

Rheumatoid arthritis (RA) is a chronic inflammatory disease that leads to joint destruction and bone erosion. Even if many treatments were developed with success in the last decades, some patients fail to respond, and disease chronicity is still a burden. Mechanisms involved in such resistance may include molecular changes in stromal cells. Other explanations can come from observations of tenosynovial giant cell tumor (TGCT), first considered as an inflammatory arthritis, but with unusual neoplastic features. TGCT leads to synovium hypertrophy and hyperplasia with hemosiderin deposition. It affects young adults, resulting in secondary osteoarthritis and increased morbidity. TGCT shows clinical, histological and genetic similarities with RA but affecting a single joint. However, the monoclonality of some synoviocytes, the presence of translocations and rare metastases also suggest a neoplastic disease, with some features common with sarcoma. TGCT is more probably in an intermediate situation between an inflammatory and a neoplastic process, with a main involvement of the proinflammatory cytokine CSF-1/CSF1R signaling axis. The key treatment option is surgery. New treatments, derived from the RA and sarcoma fields, are emerging. The tyrosine kinase inhibitor pexidartinib was recently FDA-approved as the first drug for severe TGCT where surgery is not an option. Options directly targeting the excessive proliferation of synoviocytes are at a preclinical stage.

## 1 Introduction

Rheumatoid arthritis (RA) is an inflammatory disease that primarily affects the joints. The aggressive synovitis observed is the consequence of synoviocyte hyperplasia, neoangiogenesis, and local infiltration by immune cells that produce pro-inflammatory cytokines (1, 2). Despite the development of new treatments, disease chronicity is still unresolved and some patients fail to respond to biologics. Molecular changes within stromal cells and lymphocyte-stromal cell interactions may be involved in these mechanisms of resistance (3). Other explanations can come from observations of tenosynovial giant cell tumor (TGCT), also called pigmented villonodular synovitis (PVNS), which is an inflammatory arthritis with neoplastic features.

TGCT is a rare disease characterized by massive proliferation of the synovium of joints, tendon sheaths, and bursa and affects mainly young adults. TGCT can be localized or diffuse, intra or extra-articular and leads to joint pain and swelling with bloody effusion or to a painful soft-tissue mass (4, 5). Key pathologic features combine a proliferative process in synovial tissue with hemosiderin deposition which results from the proliferation of stromal cells, accumulation of mononuclear cells and multinuclear giant cells (GCs) that are fully differentiated osteoclasts (5–7). The appearance of diffuse TGCT (dTGCT), also called pigmented villonodular synovitis (PVNS), is that of a tumor with increased number of mitoses, very large size of some cells ranging from 25 à 40 µm in diameter, as in malignant tumors (5). These histologic features are correlated with the imaging findings, which show joint effusion, tumors, and bone destruction (8). TGCT etiology remains uncertain, but this disease shares many characteristics with RA-related inflammation (9). In addition to this inflammatory process, TGCT shows neoplastic features with clonal cytogenetic abnormalities, and even, rare cases of metastasis, as in sarcoma (10, 11). Given this balance between an inflammatory and a neoplastic process, care and treatments have been developed from options already used in these two fields.

The purpose of this review is to discuss the position of TGCT between RA and sarcoma on different aspects. The first part will describe the clinical aspects. Then, histopathological lesions and genetic alterations will be analyzed. The final part will outline current treatments of TGCT and the new promising strategies arising from both RA and sarcoma management, with the importance of a multidisciplinary approach.

## 2 TGCT Clinical Spectrum: From RA to Sarcoma

**Figure 1:**
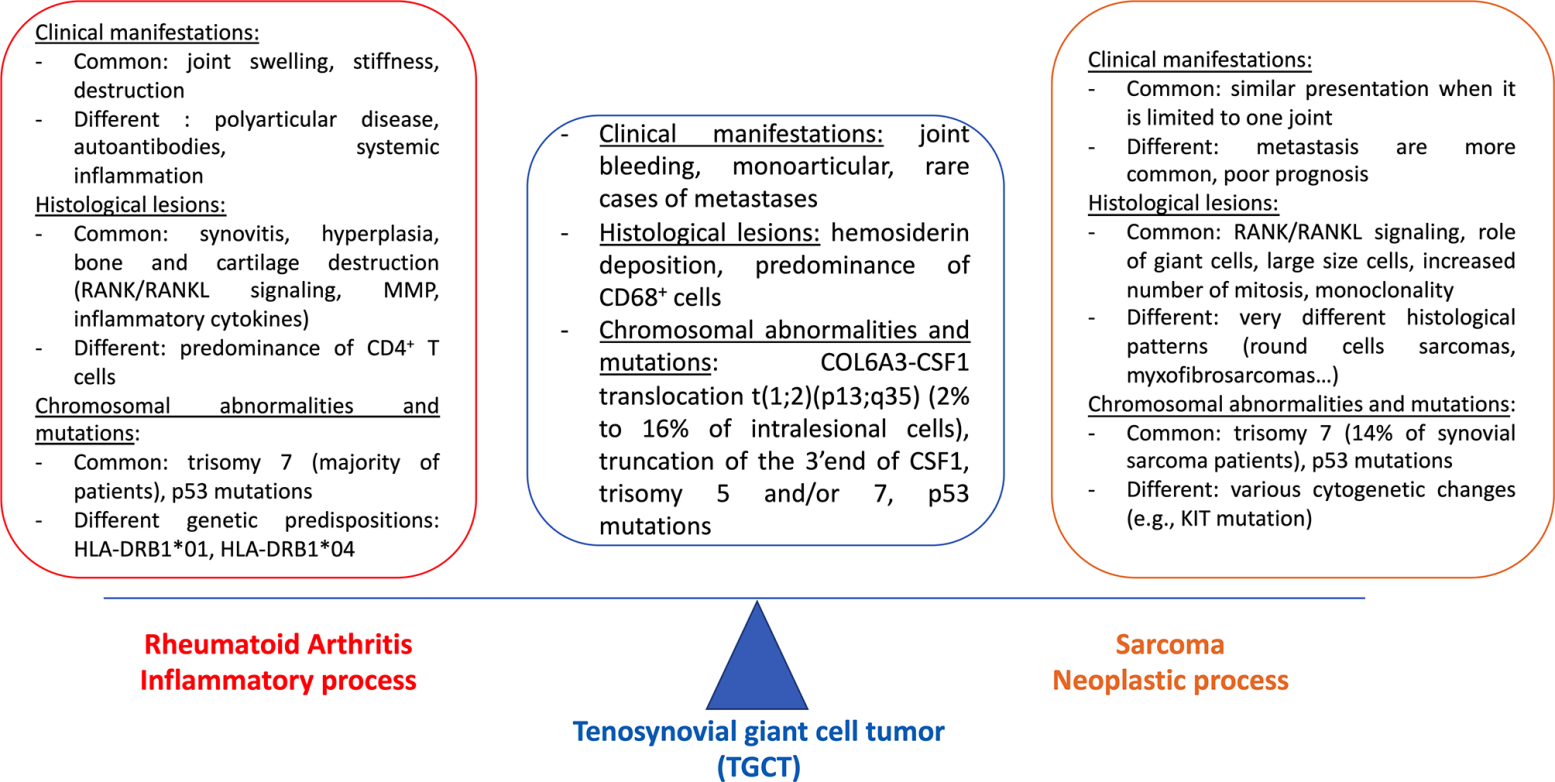
Tenosynovial giant cell tumor (TGCT), an intermediate between rheumatoid arthritis (RA) and sarcoma. Tenosynovial giant cell tumor (TGCT) is generally a monoarticular inflammatory disease characterized by joint bleeding. Metastases are very rare. Histological analysis reveals hemosiderin deposition and a predominance of CD68^+^ cells. Collagen-6 A3 (COL6A3)/CSF1 translocation t (1;2) (p13;q35) is present in two to 16% of intralesional cells, 3’end truncation of CSF1 seems to be even more frequent. Trisomy 5 and/or 7, p53 mutations are possible. TGCT has been often considered as an inflammatory disease, close to RA. Reasons include similar clinical manifestations (joint swelling, destruction), histopathological lesions (synovitis, hyperplasia, bone, and cartilage destruction) and chromosomal aberrations (trisomy 7, p53 mutations). However, some differences exist as RA is a polyarticular disease with autoantibodies and systemic inflammation, with HLA-DR genetic predisposition. There is a majority of CD4^+^T cells in RA synovium. There is an HLA-DR genetic predisposition. TGCT also has neoplastic characteristics. Clinical presentation can be that of a single joint sarcoma. The monoclonality of some cells is also in favor of a neoplastic process, other common histological lesions include the role of giant cells, large size cells, and an increased number of mitosis. Trisomy 7 and p53 mutations are found both in TGCT and in sarcoma. However, metastasis are more common in sarcoma with poor prognosis.

TGCT is a locally aggressive synovial disease with inflammatory pattern sharing common aspects with RA. However, TGCT appears also like a neoplastic process because of some similarities with sarcoma. In this part, similarities and differences of the clinical manifestations of these three conditions will be developed (**Figure 1**).

### 2.1 TGCT

TGCT is characterized by an overgrowth of the synovium of joints, less frequently of tendon sheaths, or bursa. It affects mostly young adults with a modest female predominance (12). Two anatomical forms are described according to the spread of the disease and both can arise from intra or extra-articular synovial tissue. Roughly 90% of TGCT are localized (4). The localized form of TGCT is characterized by a single mass (± 2cm), well circumscribed in the synovium (13, 14). TGCT is considered as diffuse when it affects one compartment, or the entire synovium of a given joint and is sometimes called dTGCT. It affects mainly large joints (>5cm) with the knee as the most common localization, followed by hip, shoulder, and ankle. The diffuse form can be multinodular and is considered as a more aggressive lesion with a high incidence of recurrence and of joint destruction. Such destruction is in part linked to bleeding itself, as seen in hemophilia (4, 15). The clinical manifestations of TGCT depend on the location and the extent of the disease. In localized TGCT form, mechanical symptoms such as locking or joint instability are more prominent (4, 13). The diffuse form is characterized by mild pain with restricted mobility related to joint effusion and swelling (4, 12). Joint bleeding is very suggestive of TGCT (4). Spinal TGCT is rare with possible neurological consequences (16).

As these symptoms are rather unspecific, there is often a delay in diagnosis. The key exam is MRI that defines lesion extension and bone involvement. MRI signal intensity depends on lesion characteristics (hemosiderin deposits, synovial proliferation, joint effusion, etc.…). Conventional radiographs are less contributive and show rather late joint damage. Although not systematic, pre-operative biopsy can help to confirm diagnosis showing synovial hypertrophy and hyperplasia with hemosiderin deposition (14).

Diffuse TGCT has a higher recurrence rate than localized TGCT, with a more rapid joint destruction, number of surgical events, and secondary osteoarthritis (OA). Therefore, the resulting quality of life is decreased in TGCT patients compared with the general population (14).

### 2.2 RA

RA, one of the most prevalent joint diseases, can rarely start as a mono-articular arthritis. It can constitute, to some extent, a differential diagnosis for TGCT. This atypical presentation can delay RA diagnosis. In terms of epidemiology, RA affects much more women than TGCT, and TGCT begins earlier than RA. The role of estrogen on the immune system is systemic and may account, partially, to the different sex ratio observed between RA and TGCT. Indeed, RA is associated with systemic inflammation contrary to TGCT which is restricted to a single-joint. TGCT can follow traumatisms and occurs earlier (12, 17, 18). Clinical manifestations from both RA and TGCT include joint swelling, stiffness, and later destruction. RA affects several joints with a symmetrical presentation while TGCT is generally mono-articular and associated with joint bleeding (4, 17, 18). But RA can start as a mono-articular disease, and biopsy will clarify the diagnosis. As opposed to TGCT, RA is often characterized by abnormal laboratory tests with positive rheumatoid factor (RF), the presence of anti-citrullinated protein antibodies (ACPA) and elevated c-reactive protein (CRP), or erythrocyte sedimentation rate (ESR) (17). Both diseases affect quality of life. In addition, RA is also associated with premature cardiovascular events, osteoporosis, and risk of lymphoma (19).

### 2.3 Sarcoma

Another differential diagnosis of TGCT is sarcoma. Sarcomas are a heterogeneous group of rare mesenchymal malignancies with over 150 subtypes (20, 21). They arise from soft tissue (75% of cases), visceral tissue (15%) or bone (10%). Clinical manifestations depend on sarcoma subtype of sarcomas and the anatomic site. As opposed to TGCT that is rarely life-threatening, metastatic, and refractory sarcomas have a poor prognosis (20–22).

Among soft tissue sarcomas, intra-articular sarcomas have rather similar MRI characteristics to localized TGCT making differential diagnosis difficult. Synovial sarcoma (SS) is the most frequent intra-articular sarcoma (23, 24). The term “synovial” in SS only reflects histopathological similarities with synovial tissue but the tumor *per se* does not origin from synovium. Its occurrence within a joint is very rare and rather affects soft tissues around large joints, especially the knee. Compared with TGCT, patients with sarcoma tend to be younger and of male gender, lesions are usually large, with a small effusion, and calcifications (24). The main difference between TGCT and SS is the high potential for metastasis of SS mainly in lungs. There are rare proven cases of metastasis of TGCT (25).

To conclude, TGCT shares common features with both RA and sarcoma. Clinical manifestations, laboratory tests and MRI help to differentiate these diseases, but biopsy remains the most powerful tool.

## 3 TGCT, a Histologic Arthritis With Neoplastic Features

Considering that TGCT is at the interface of RA and sarcoma, its pathophysiology is first described, and then compared with each other disease (**Figure 1**).

### 3.1 TGCT

TGCT is characterized by a synovial hypertrophy and hyperplasia with hemosiderin deposition (9). The hypertrophy consists of villonodular protrusions of synovium and of papillary, or villous projections. Villi stroma is infiltrated by cells with hemosiderin pigments from bleeding. Hyperplasia results from the proliferation of multinuclear GCs and synovial fibroblasts, or synoviocytes with accumulation of mononuclear cells (5–7). Most of these mononuclear cells stain positive for CD68, a marker of macrophages. GCs are also positive for CD68 staining and for tartrate-resistant acid phosphatase (TRAP) and calcitonin-receptor (CTR), which are markers of osteoclasts (6, 7). Osteoclast-like GCs are formed from CD14^+^ monocytes and are capable of lacunar resorption, while CD14^-^ monocytes support osteoclast formation and express osteoclastogenic factors [receptor activator of nuclear factor ligand RANKL and macrophage colony stimulating factor (M-CSF), also known as colony stimulating factor (CSF-1)] (26). CSF1 receptor (CSF1R) is expressed by mononuclear cells and GCs (27). Cytokines such as tumor necrosis factor (TNF)α, interleukin (IL)-6, and IL-1, derived from monocytes and stromal cells, activate osteoclasts, participate in bone resorption and stimulate matrix metalloproteinases (MMP) production, inducing cartilage matrix breakdown (6, 28). In addition, defects of apoptosis in monocytes and GCs are associated with a strong expression of p53 (6, 29, 30). Some forms of TGCT have a more aggressive phenotype with very large size cells, large nucleoli structures, and increased number of mitosis (5).

### 3.2 TGCT and RA

RA synovitis is associated with hyperplasia of synoviocytes, neoangiogenesis, and local infiltration by immune cells (1). Macrophages that are present in the synovium produce pro-inflammatory cytokines like TNFα, IL-6, or IL-1. These cytokines stimulate synoviocyte hyperproliferation and aggressiveness. This abnormal proliferation is a common feature of both TGCT and RA (1, 9). As described for TGCT, RA synovium shows increased levels of MMP that participate to cartilage damage. Synoviocytes are also involved in bone erosion through the production of RANKL which promotes osteoclast differentiation. Moreover, recent results suggest that RA synovial cells behave and perform as osteoclast-like cells in an IL-6/IL-6R-driven metabolic reprogramming manner and then secrete increased levels of RANKL (31). Finally, apoptosis is impaired in RA synoviocytes showing p53 mutations, as found in cancer (32).

While there are common points between TGCT and RA synovium, the pathology picture is in fact rather different, with important differences. For instance, the predominant cell type in TGCT is CD68^+^ (6). In RA, about half of the synovial sublining cells are CD4^+^ memory T cells. These cells can also form germinal centers where antibodies are produced (2). For all these reasons, TGCT has long been considered as an inflammatory disease, looking like but in fact different from RA.

### 3.3 TGCT and Sarcoma

As described above, TGCT share similar features with sarcomas, especially the intra-articular SS (24). Cell morphology in sarcoma can be various with spindle, epithelioid, and round cells (33). As in TGCT, hemosiderin, osteoclast-like GC and synovitis can also be found in sarcoma (23, 24, 34). RANK/RANKL signaling is also involved in osteosarcoma leading to bone destruction (35). Moreover, CSF1 is expressed by malignant cells in leiomyosarcoma and is associated with poor prognosis. CSF1R is expressed both by macrophages and cancer cells (36).

The difficulty to distinguish TGCT and sarcoma relies on the fact that 1% to 3% of TGCT can undergo true malignant degeneration and become GC-rich osteosarcomas (22). The evolution of TGCT lesion is possible towards a more aggressive phenotype (large size cells, number of mitosis, etc.), with poor prognosis (5).

Sarcomas can show epithelioid morphology, as in TGCT. However, many other histologic patterns are described with round cells sarcomas, myxofibrosarcomas, and undifferentiated pleiomorphic sarcomas that are strictly different from TGCT (33).

Therefore, observations from histological examinations reveal that TGCT lesion is closed to RA synovitis and, to some extent, to sarcoma lesion (**Figure 1**). Results from genetic studies are now presented.

## 4 Genetic Similarities and Differences Between TGCT, RA and Sarcoma

In addition to histopathological similarities and differences, TGCT, RA and sarcoma share some genetic and molecular aspects.

### 4.1 TGCT

Several genetic and chromosomal alterations have been identified in TGCT. Only a few cells in a lesion have these changes. Some of these TGCT cells are aneuploid and exhibit a trisomy 5 and/or 7 suggesting clonality, and a neoplastic origin (11, 37–39). Trisomy 7 could confer a proliferative advantage to the affected cells. Indeed, chromosome 7 contains genes like epidermal growth factor (EGFR), which is involved in epithelial tissue development and homeostasis. Synovial mesenchymal stem cells also harbor a trisomy 7. One can suppose that there is a link between the synovial hyperproliferation, EGFR pathway and TGCT (38, 40). The effect of trisomy 5 is uncertain. These trisomies are found both in primary, recurrent, and metastatic lesions (39).

Cytogenetic analysis has shown that a small fraction of TGCT cells has a specific translocation t(1;2)(p13;q35) that involves CSF1/M-CSF1 gene, on 1p13, and collagen 6A3 gene, on 2q35. This translocation is present in the majority of TGCT. The resulting fusion protein leads to CSF1 (M-CSF) overexpression that increases and attracts non-neoplastic monocyte-like inflammatory cells that express CSFR1/M-CSFR. However, the translocation is present only in two to 16% of intralesional cells meaning that most cells are non-neoplastic ones and recruited solely through the local overexpression, and production of CSF1. Recently, the loss or the replacement of the negative regulatory elements in the 3’end of CSF1 was described with FISH and RNAseq methods as even more frequent than COL6A3-CSF1 fusion. Overall, these alterations lead to the formation of a tumor mass with CSF-1/CSF-1R as a central mechanism of tumorigenesis (10, 41, 42). As mentioned above, CSF-1 and RANKL expressed at high levels, allow osteoclast-like multinucleated cell accumulation resulting in bone resorption and joint destruction.

### 4.2 TGCT and RA

Some of these features have also been identified in RA. Presence of trisomy 7 has been detected in RA synovial cells in a small size study showing such trisomy 7 in six of the seven studied cases. The proportion of cells with this change correlates with overgrowth of synoviocytes (43). Similar results apply to synovial fluid cells where trisomy 7 occurs in 53% of patients and in a variable percentage of cells (from 23 to 48%) (44). *In vitro* studies in RA synovial cells showed that trisomy 7 leads to synovial hyperproliferation (45). As opposed to TGCT, trisomy 5 has not been found in RA (44). In both diseases, these chromosomal aberrations may play a role in enhancing synoviocyte proliferation leading to cartilage and bone destruction.

The CSF-1/CSF-1R overexpression is not unique to TGCT and is found in RA. However, the pattern of expression is very different with diffuse staining in TGCT and CSF1 translocation is rather constant. In RA, CSF-1/CSF-1R expression is only localized in the synovial lining and the translocation is not found (46).

Contrary to TGCT, RA has a strong genetic component and twins show a disease concordance of 12-15%. RA susceptibility variants include specific class II human leukocyte antigen (HLA), especially HLA-DRB1*01 and HLA-DRB1*04. These variants are associated with an increased risk of developing RA. Some of RA susceptibility genes are associated with increased severity (2).

### 4.3 TGCT and Sarcoma

Common chromosomal alterations exist in both diseases. For instance, trisomy 7 was found in 14% of SS, in 9% of Ewing’s sarcomas (ES), and 2% of myxoid liposarcomas (47). Even if translocation t(1;2)(p13;q35) that involves CSF1 gene is not commonly found in sarcoma, ES proteins bind to the proximal elements of the CSF1R promoter (48). The expression of CSF-1/CSF-1R is variable in soft tissue sarcomas (46).

Sarcomas can be classified according to cytogenetic changes and the complexity of their karyotypes. Approximately half of sarcomas are regarded as “sarcomas with complex genomics”. Whereas the other half may be referred to as “sarcomas with simple genomics”, characterized by recurrent genetic alterations (translocations, mutations and amplifications). Other molecular alterations include mutation of KIT proto-oncogene receptor tyrosine kinase. This mutation occurs in some sarcomas and this has been the basis for the use of tyrosine kinase inhibitors (TKI) (33).

**Figure 2:**
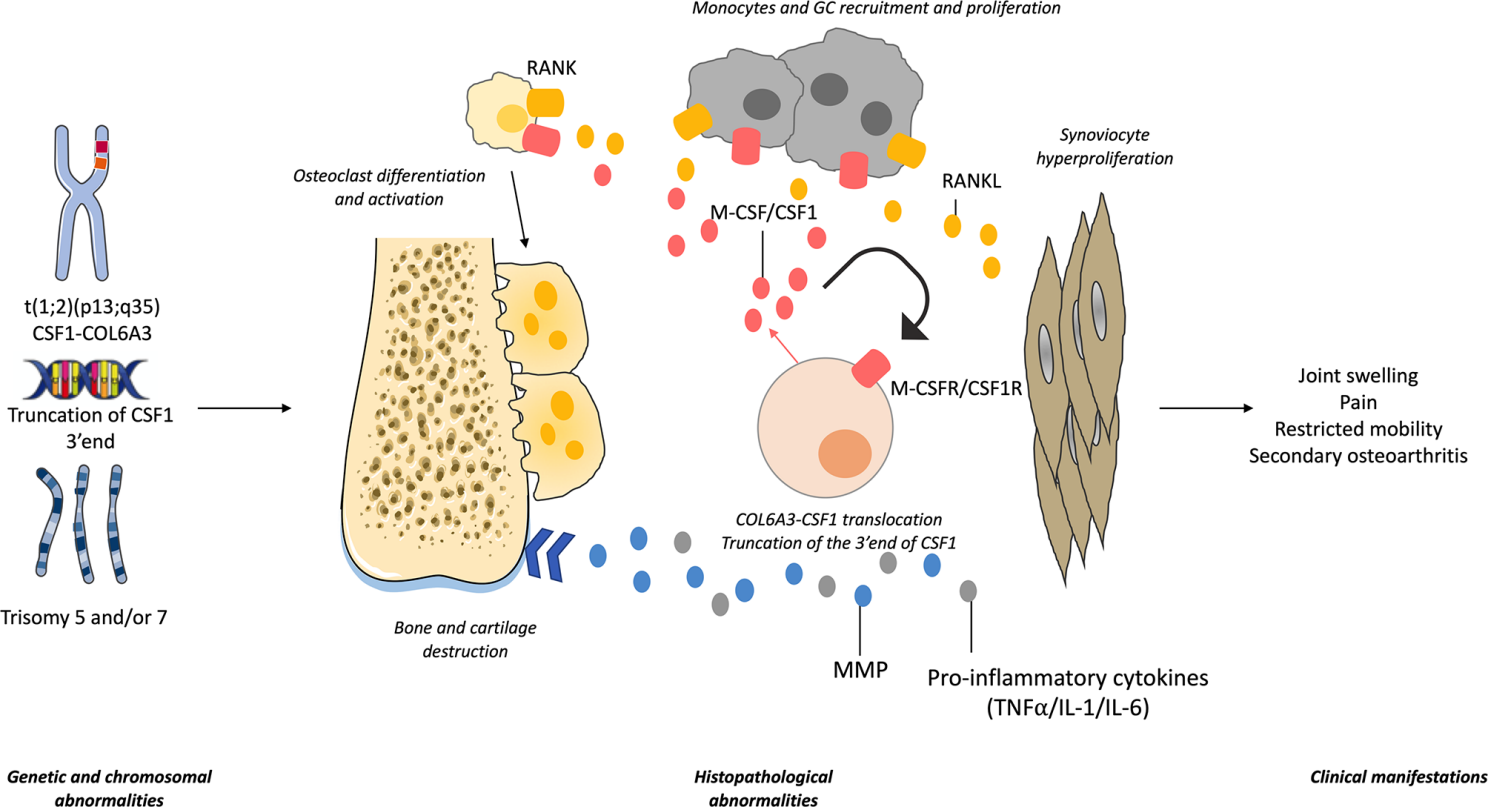
Tenosynovial giant cell tumor (TGCT) pathophysiology. Genetic and chromosomal aberrations have been found in TGCT. It includes the translocation t(1;2) (p13;q35) involving CSF1/M-CSF and collagen-6 A3 (COL6A3) genes or the truncation of CSF1 3’end. Trisomies 5 and/or 7 are also found in some cells. Cells with the translocation produce high levels of CSF1 which acts in autocrine and paracrine manner. It allows the recruitment of monocytes, multinuclear giant cells (GCs) and promotes osteoclast differentiation. The receptor activator of nuclear factor kappa-B (RANK)/RANK ligand (RANKL) pathway induces similar effects. The presence of pro-inflammatory cytokines (tumor necrosis factor (TNF)α, interleukin (IL-1, IL-6) and matrix metalloproteinases (MMP) triggers synoviocyte proliferation, and joint destruction. Clinical manifestations include joint swelling and pain, restricted mobility and in some cases, secondary osteoarthritis.

To conclude this part, similarities and differences in genetic alterations that have been described between TGCT, RA and sarcoma are summarized in **Figure 1**. The pathophysiology of TGCT, from genetic and chromosomal alterations to clinical manifestations, is summarized in **Figure 2**.

## 5 Treatment

**Figure 3:**
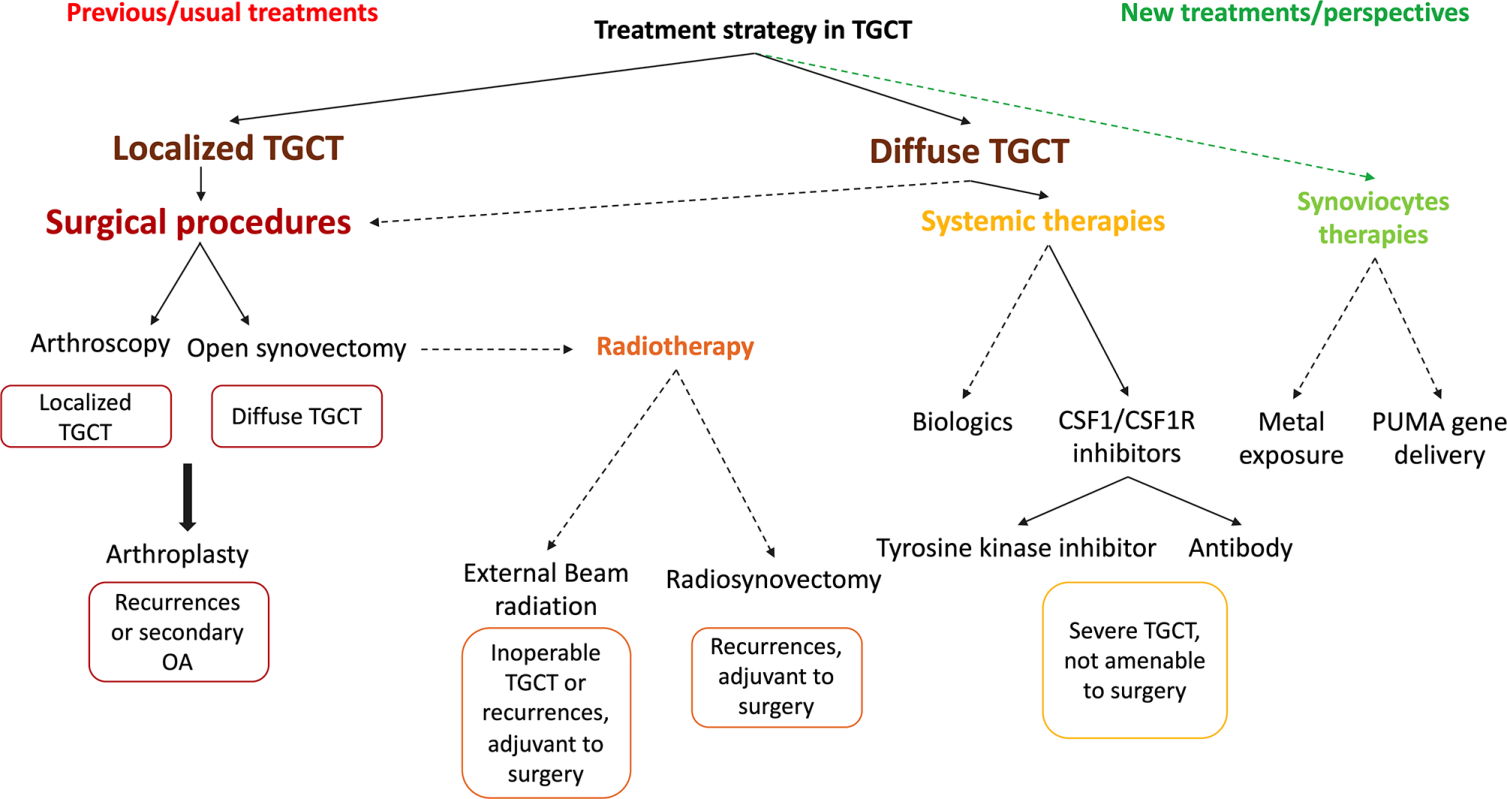
Treatment strategy in tenosynovial giant cell tumor (TGCT). Treatment strategy mainly relies on the type of TGCT. Localized forms TGCT are eligible for surgical resection, which can allow a full control of the disease. There is no clear consensus on the technique to be used. Arthroscopy is preferred for these forms of TGCT. If surgery is performed in the case of diffuse TGCT, open synovectomy is preferred to limit the risk of recurrence. However, regarding the risk of recurrence, diffuse TGCT are prone to secondary osteoarthritis and arthroplasty is sometimes necessary at a young age. Radiotherapy is less used today and includes external beam radiation (EBR) and radiosynovectomy. Systemic therapies have been recently developed and are particularly adapted to diffuse form of the disease. Anti-cytokine biologics have tested only in individual cases. The real improvement comes from CSF1/CSF1R inhibitors and especially with tyrosine kinase inhibitors. Pexidartinib is the first systemic FDA-approved treatment for severe TGCT, when surgery is not an option. As perspectives, local intra-articular therapies targeting synoviocytes could be of interest with intra-articular metal exposure of cadmium or p53 upregulated modulator of apoptosis (PUMA) gene delivery.

For many years, the key and only treatment was surgical excision, but without any consensus on the most appropriate type of surgery. These methods were also used in RA (49). The high rate of recurrences (10% in localized TGCT, 40% in dTGCT) and the need of multiple episodes of surgery have indicated the need of new systemic or intra-articular therapies (50). As TGCT is mainly a local disease, the development of local therapies may represent the best option. A multidisciplinary approach is needed to manage TGCT, and treatment strategies are summarized in **Figure 3**. General practitioners, rheumatologists, physical medicine and rehabilitation doctors, orthopedic surgeons, and oncologists should be involved to choose the most appropriate strategy to each patient. Regarding the last treatments developed in TGCT, an algorithm is proposed in **Figure 3** based on the presentation of the disease and the risk of recurrence. Patient needs and preferences must be included in the management (51).

### 5.1 Current Treatments

#### 5.1.1 Surgical Procedures

Surgical procedures include different types of techniques: arthroscopic or open surgical excision with partial, or complete synovectomy. Usually, arthroscopic synovectomy is used for localized forms while open total synovectomy is common for dTGCT, but there is no clear consensus (49). Localized TGCT are usually solved with surgical excision, which makes it the first-line treatment in this form of the disease (52). In dTGCT, there is a high risk for local recurrent disease and for postoperative complications. Risk factors for such complications are unknown to date. If surgery alone is likely to be insufficient, a multidisciplinary evaluation is absolutely needed (50). It might be difficult to differentiate manifestations linked to inflammation, with variation from day to day, to those linked to joint destruction, very sensitive to exercise.

##### 5.1.1.1 Arthroscopy

Arthroscopy is a minimally invasive procedure with a particularly low risk of complications. Compared with open surgery, arthroscopy allows faster rehabilitation, less postoperative pain, can be performed as a day care but can be technically demanding (53).

Arthroscopic synovectomy appears as the best method to treat localized TGCT with low rate of recurrences (0-6%) (54, 55). The choice between partial or complete arthroscopic synovectomy depends on disease extension. For local disease, a synovectomy limited to the lesion can be sufficient (55). Rather similar conclusion applies for diffuse knee TGCT, but a complete synovectomy should be preferred to lower the risk of recurrence. However, it should be remembered that a so-called complete synovectomy is never complete, specifically for posterior lesions. Most of the studies are on knee TGCT, but with less results for other joints (shoulder, hip, or ankle) (54–56).

##### 5.1.1.2 Open Synovectomy

Open synovectomy is an open surgery that can be performed in all joints. This technique shows advantages as the easiest to perform but also drawbacks with an increased patient morbidity, a longer rehabilitation, and an overall risk of post-operative complications higher than arthroscopy (53).

This method is generally chosen for dTGCT where recurrence rate is more important. Such open procedure is supposed to allow a more complete synovectomy. For the knee, a two-stage approach (anterior and posterior) appears as a safe treatment option with an average recurrence rate lower than for arthroscopy (55). There are less studies on other joints, but open complete synovectomy seems to be the best-suited method for dTGCT.

Open synovectomy and arthroscopy are often associated to control dTGCT. This combined synovectomy results in a better outcome for diffuse knee TGCT.

##### 5.1.1.3 Arthroplasty

Arthroplasty is not a first-line treatment of TGCT but remains necessary when patients have many recurrences or refractory disease leading to OA. Indeed, chronic joint inflammation and bleeding combined with the multiple surgeries lead to secondary OA. This requires joint replacement at a relatively young age (49). Total arthroplasty is associated with a low rate of local recurrence and clear improvement in function but the rate of post-operative revisions in these young patients is elevated.

#### 5.1.2 Radiotherapy

Two forms of radiation therapy have been used for TGCT: external beam or intra-articular radiotherapies. These two approaches improved local disease control and were mainly considered as adjuvant therapies but are rarely used today (56).

##### 5.1.2.1 External Beam Radiation

External beam radiation (EBR) has two main indications in TGCT with inoperable disease or as an adjuvant treatment when recurrence occurred, or if surgery was incomplete. Better results are described when EBR is used just after synovectomy with a low recurrence rate. The benefit of EBR is more important when EBR is performed within three months after surgical knee synovectomy and not used to treat a recurrence that has already developed. No major adverse effects have been reported. This radiation therapy is not recommended for hand and foot lesions (55).

##### 5.1.2.2 Radiosynovectomy

Radiosynovectomy, also called isotopic synoviorthesis, consists in the intra-articular injection of radioactive products. The anti-inflammatory action consists of synoviocyte radio-lesion and necrosis with no risk of extra-articular migration and degradation of the surrounding tissues. This technique has been used a lot in RA before the use of biotherapies (57). As for EBR, this approach is mainly proposed just after synovectomy. Despite the relative low number of trials using this technique, intra-articular injections of isotopes appear safe and to reduce the recurrence rate. However, the inherent risk of radioactive isotopes has strongly limited its indications (12, 54, 55).

These local treatments are often far from always active to control diffuse and recurrent TGCT. However, they are suited to local forms of the disease.

### 5.2 New Therapeutic Strategies

As for the clinical presentation and pathogenesis, new treatments are emerging, often from the cancer and RA fields. As discussed above, surgical procedures do not prevent the risk of recurrence in diffuse TGCT, where systemic therapies have a growing interest. Other local therapies are discussed as perspectives.

#### 5.2.1 Targeted Therapies

CSF-1 overexpression by TGCT clones has led to the option to target the CSF1/CSF1R-signaling axis (22). Two strategies have been developed to block CSF1R axis: blockade of the tyrosine kinase activity of CSF-1R with small inhibitory molecules (TKI), or use of monoclonal antibodies targeting CSF1-R.

##### 5.2.1.1 Tyrosine Kinase Inhibitors (TKI)

TKI are widely used in oncology, notably in sarcomas. Imatinib that blocks CSF1R activation was the first to be used in TGCT, with induction of a complete response in a case of recurrent elbow TGCT (41). A relapse occurred when treatment was stopped and a second complete remission was observed after reintroduction (41, 58). A larger retrospective study confirmed these results with 20/27 patients achieving tumor control and the objective response was recorded in nearly 20% (5/27) of patients (59). In addition, nilotinib, a multi-target TKI, was assessed in progressive or relapse TGCT without surgical alternative in a phase 2 trial. Treatment led to tumor control in 92·6% of patients at 12 weeks and long-lasting disease stabilization in more than half of patients. However, almost all patients experienced side effects with 11% (6/56) having at least one grade 3 treatment-related adverse event (60).

Pexidartinib, another TKI with a greater specificity for CSF1-R, was tested in TGCT. To confirm results obtained in a phase 1 study, pexidartinib was compared to placebo in advanced TGCT when surgery was not recommended (61, 62). In this randomized, phase 3 study, 39% (24/61) of the treated patients achieved overall response at week 25 *vs*. 0% (0/59) in the placebo group. Results were then confirmed with an overall response increased to 53% at 22 month-median follow-up. However, 44% (27/61) of patients treated with pexidartinib presented grade 3 or 4 adverse events *vs*. 12% (7/59) in the placebo group with a higher incidence of hepatic adverse events (62). Two types of hepatoxicity have been described. The most common is the elevation of transaminases, which is dose dependent and sensitive to dose reduction. The other form is not dose related and linked to a vanishing bile duct syndrome that can be very severe. Other adverse events include changes in hair and skin color, nausea, fatigue. Based on these results, pexidartinib has been approved by the FDA in 2019 for adults with symptomatic TGCT associated with severe morbidity or functional limitations, in which surgery is not an option. Pexidartinib is now the preferred regimen in this indication, before imatinib. It is associated to a warning box because of liver toxicity, with mandatory frequent liver enzyme testing. However, this toxicity has prevented EMA registration (63). In case of resistance or intolerance to TKI, switch from one to another may constitute an alternative (60, 64). Other trials are on-going (NCT04526704, NCT01207492, NCT04488822, NCT03069469) (65).

##### 5.2.1.2 Anti-CSF1/CSF1R Monoclonal Antibodies

Another way to block CSF1/CSF1R axis is to use anti-CSF1R antibodies. Emactuzumab (66–68) and cabiralizumab (69) have been developed and showed encouraging results, mainly in phase 1 trials. Five of the seven patients treated with emactuzumab achieved partial responses and clinical activity correlates with a reduction of macrophages and CSF-1R^+^ cells in matching tumor biopsies (66). Objective responses were noted in 86% (24/28) of patients treated with emactuzumab in a larger trial. Common adverse events were facial edema, asthenia, and pruritus (67). Phase 2 study was published for this monoclonal antibody in TGCT showing that the optimal biological dose was more than two-fold lower than the highest dose tested in the trial (67, 68, 70). Monoclonal antibody targeting CSF1 (lacnotuzumab) is currently tested in phase 2 trial (NCT01643850), regarding promising results obtained in four patients (71). Other trials studying the efficacy of these drugs are on-going (NCT02471716).

Overall, randomized trials are warranted to confirm these promising results in TGCT and pexidartinib now offers a relevant treatment option for selected patients (72). In the future, these systemic therapies may be considered as neoadjuvant treatment to decrease recurrence rates after surgery (73). Another option to be fully tested is taking advantage of the effects of these drugs on tumor volume to use them first and allow easier surgery.

Beyond the targeting of the CSF1/CSFR1 axis, results from histopathological analysis allow to outline some perspectives for developing new treatments in TGCT. Many of them come from RA.

#### 5.2.2 Anti-Cytokine Biologics

Pro-inflammatory cytokines contribute to both RA and TGCT and their inhibitors have been used for 20 years in RA (28, 74). The association of methotrexate and the anti-TNFα antibody infliximab in a refractory case of knee TGCT resulted in marked clinical improvement (75). Intra-articular injections of etanercept, another TNFα blocker, used concomitantly with disease modifying anti-rheumatic drugs have been used in two patients with severe knee TGCT. Without adverse events, a marked improvement of disease activity was obtained for both patients and ultrasound imaging confirmed the regression of synovial proliferation (76). Similar results were obtained with intra-articular injection of adalimumab with a marked clinical improvement and MRI regression of knee lesion (77). These isolated cases are of interest, but adequate clinical trials are obviously needed.

#### 5.2.3 Local Targeting of Synoviocytes

TGCT and RA are linked to an excessive and uncontrolled proliferation of synoviocytes (9). Two methods will be described below with the promising use of metals and the effect of p53 upregulated modulator of apoptosis (PUMA) gene delivery into *in vitro* and *in vivo* models of RA, that could be of interest in TGCT.

##### 5.2.3.1 Intra-Articular Metal Exposure

Several metals can modulate inflammation and cell viability. Zinc (Zn) and cadmium (Cd) share similar characteristics and transporters but their actions on the immune system is often opposite. Zn is necessary for a normal immune response whereas Cd has deleterious effects on cells (78, 79). The pro-apoptotic properties of Cd have been tested in RA models. Exposure to Cd induces massive killing of synoviocytes cultured in inflammatory conditions. Similar results are described in co-cultures of blood mononuclear cells and RA synoviocytes, and in RA synovial explants. These observations were confirmed *in vivo* in a model of arthritis (80). Cd intra-articular injection improves clinical scores, reduces inflammation, and joint destruction in rats without harmful toxicity (79). Therefore, intra-articular treatment with Cd could be applied to control TGCT (81).

##### 5.2.3.2 PUMA Gene Delivery to Synoviocytes

Regarding TGCT pathophysiology, the *in situ* targeting of synoviocyte hyperproliferation and inflammation would be of interest. A model was developed in RA with the induction of synoviocyte apoptosis through gene targeting. The pro-apoptotic gene PUMA combined with an new adenovirus-baculovirus complex vector (82) has shown a massive killing effect of synoviocytes and reduction of inflammation, and destruction in an *in vivo* model of RA (83). Therefore, intra-articular gene delivery could constitute another option for TGCT. In both options, the small amount of product needed for local administration would limit systemic toxicity.

## 6 Conclusion

TGCT is a cause of disability in young adults because of its high level of recurrence and of bone destruction, aggravated by recurrent bleeding. Its pathophysiology is now better understood. Genetic and chromosomal aberrations induce synovial hypertrophy and hyperplasia. TGCT shares common features with RA and sarcoma but with differences.

The multiple presentations of the disease are the basis for treatment strategies. Usual treatments rely on surgical procedures, but limited results are affected by the high recurrence rate. New treatments are developed and inspired by those of RA and sarcoma. Systemic therapies targeting the CSF1/CSFR1 axis show promising results and pexidartinib is now FDA-approved for TGCT but managing liver toxicity is critical. Local treatments targeting synoviocytes are also under development. Overall, a multidisciplinary approach is essential for TGCT management. A such approach could also serve to understand better unresolved mechanisms of resistance in RA.

## Data Availability Statement

The original contributions presented in the study are included in the article/supplementary material. Further inquiries can be directed to the corresponding author.

## Author Contributions

MR and HF: writing and figures. PM: concept and proof reading. All authors contributed to the article and approved the submitted version.

## Funding

This work has been supported by the OPeRa IHU program, and the Institut Universitaire de France. PM is a senior member of the Institut Universitaire de France. MR is supported by the Ecole de l’INSERM Liliane Bettencourt Programme.

## Conflict of Interest

PM has a patent for the use of PUMA gene therapy in arthritis.

The remaining authors declare that the research was conducted in the absence of any commercial or financial relationships that could be construed as a potential conflict of interest.

## Publisher’s Note

All claims expressed in this article are solely those of the authors and do not necessarily represent those of their affiliated organizations, or those of the publisher, the editors and the reviewers. Any product that may be evaluated in this article, or claim that may be made by its manufacturer, is not guaranteed or endorsed by the publisher.
